# Regenerative Rehabilitation and Stem Cell Therapy Targeting Chronic Spinal Cord Injury: A Review of Preclinical Studies

**DOI:** 10.3390/cells11040685

**Published:** 2022-02-16

**Authors:** Syoichi Tashiro, Masaya Nakamura, Hideyuki Okano

**Affiliations:** 1 Department of Rehabilitation Medicine, Keio University School of Medicine, Shinjuku City, Tokyo 160-8582, Japan; 2 Department of Rehabilitation Medicine, Kyorin University School of Medicine, Mitaka City, Tokyo 181-8611, Japan; 3 Department of Orthopaedic Surgery, Keio University School of Medicine, Shinjuku City, Tokyo 160-8582, Japan; 4 Department of Physiology, Keio University School of Medicine, Shinjuku City, Tokyo 160-8582, Japan

**Keywords:** exercise, graft, neurorehabilitation, physical therapy, plasticity, regenerative medicine, transplantation, training

## Abstract

Stem cell medicine has led to functional recovery in the acute-to-subacute phase of spinal cord injury (SCI), but not yet in the chronic phase, during which various molecular mechanisms drastically remodel the tissue and render it treatment-resistant. Researchers are attempting to identify effective combinatorial treatments that can overcome the refractory state of the chronically injured spinal cord. Regenerative rehabilitation, combinatorial treatment with regenerative medicine that aims to elicit synergistic effects, is being developed. Rehabilitation upon SCI in preclinical studies has recently attracted more attention because it is safe, induces neuronal plasticity involving transplanted stem cells and sensorimotor circuits, and is routinely implemented in human clinics. However, regenerative rehabilitation has not been extensively reviewed, and only a few reviews have focused on the use of physical medicine modalities for rehabilitative purposes, which might be more important in the chronic phase. Here, we summarize regenerative rehabilitation studies according to the effector, site, and mechanism. Specifically, we describe effects on transplanted cells, microstructures at and distant from the lesion, and molecular changes. To establish a treatment regimen that induces robust functional recovery upon chronic SCI, further investigations are required of combinatorial treatments incorporating stem cell therapy, regenerative rehabilitation, and medication.

## 1. Introduction

Spinal cord injury (SCI) is a very severe condition with various sequelae represented by motor, sensory, and autonomic disorders, which have an enormous influence on psychosocial aspects of patients’ lives. There are two major classifications of SCI: traumatic SCI and non-traumatic SCI. Traumatic SCI is estimated to have an annual incidence ranging from 8.0 [1] to 246 [2] individuals per million and a prevalence ranging from 236 [3] to 1298 [4] individuals per million. Its incidence generally has two peaks, one at less than 30 years old and another at more than 60 years old. Researchers suggest this is because of the causes of injury, namely, traffic and sports accidents and falls [5,6,7]. Treatments for acute traumatic SCI are mostly limited to surgery involving re-stabilization of the spinal column and decompression of the spinal cord, as well as medical treatment with blood pressure augmentation, followed by rehabilitative treatments [8,9,10]. On the other hand, there is almost the same number of patients with non-traumatic SCI [11]. While the epidemiological data are limited, the annual incidence of non-traumatic SCI is estimated to be 6–76 individuals per million. Individuals with non-traumatic SCI are generally older and less severely injured because the causes include degenerative disc disease, spinal canal stenosis, cancer, and vascular events [12]. A review article reported that many spinal registries only include traumatic SCI or poorly capture non-traumatic SCI, meaning that data regarding rehabilitation are lacking, and only a limited number of studies have investigated their management [13]. Functional recovery is observed typically within the first 6 months after injury in the acute-to-subacute phase. In the chronic phase, no further functional improvement is generally expected [14].

The refractory state of the chronically injured spinal cord is a significant clinical concern. There are 50 times as many patients in the chronic phase than in the acute-to-subacute phase suffering from life-long impairment [15]. Several researchers have characterized the chronicity of SCI from several aspects, represented by the formation of fibrotic and glial scars [16]. Following primary injury of the spinal cord, direct mechanical damage accompanying blood–spinal barrier rupture induces massive inflammation at the lesion site. Inflammation further promotes secondary damage such as neuronal and glial necrosis and apoptosis, and Wallerian degeneration of axons located outside the initial site by inflammatory cells. These microenvironmental events occur in the acute-to-subacute phase for several weeks and are called secondary injury [17]. Low-grade inflammation persists after the subacute phase, fibrotic tissue remodeling is induced in the lesion core by fibroblast-like cells or macrophages that infiltrate from the perivascular region, and extracellular matrix components directly inhibit neural regeneration. Scar-forming reactive astrocytes densely populate the lesion margin and form a barrier-like structure, and intermediate filament proteins are expressed following inflammation. Moreover, both perilesional and distal reactive astrocytes produce chondroitin sulfate proteoglycan (CSPG) [18]. While these factors are essential to prevent spreading of persistent inflammation, they also contribute to regeneration failure of the injured spinal cord induced naturally or via regenerative treatments [19].

Stem cell-based regenerative therapy is an innovative treatment for sequelae of SCI. Significant therapeutic effects have been reported clinically and preclinically in the acute and subacute phases with various cell sources, including neural stem/progenitor cells (NS/PCs), mesenchymal stem cells (MSCs), and olfactory ensheathing cells (OECs). Researchers have elucidated various therapeutic mechanisms, including transplanted cell-mediated neuronal replacement, remyelination, and trophic support, which further induce tissue protection and enhance neuronal plasticity [20,21,22]. In contrast with the effects observed in the acute-to-subacute phase, the chronically injured spinal cord only shows a limited response to cell therapies due to the inhibitory microenvironment [9,17]. The use of stem cells as a treatment in the chronic phase of patients with SCI is controversial. This is related to concerns such as adverse events including fever, infection, sensory disturbance, and muscle weakness in general [23], and the risk of tumorigenesis with embryonic stem cells and induced pluripotent stem cell-derived cells [9,24]. Various combinatorial treatments are being investigated to achieve significant functional recovery in chronic SCI patients. While rehabilitation is generally implemented along with stem cell therapies in human patients, combinatorial treatment with rehabilitation is just beginning to be investigated in preclinical research. A combination of rehabilitation and stem cell therapy is called regenerative rehabilitation and has attracted widespread attention as a safe, feasible, and effective strategy [25,26,27]. However, to our knowledge, there is no standardized method or systematic review because this field is in its infancy. The representative beneficial effects of rehabilitation on stem cell therapy include neuroprotection of host tissue [28,29,30], induction of cell differentiation into neurons and oligodendrocytes in grafts [29,30,31], neuronal and axonal regeneration [32,33], and lumbar circuit reorganization [29,31,34,35], all of which improve functional recovery.

Although the number of studies of regenerative rehabilitation in the acute-to-subacute phase of SCI has increased, very few studies have been performed in the chronic phase [31,33,36]. In human patients, the chronic phase is 12–18 months post-injury, at which point, functional recovery plateaus. A consensus paper concerning cellular therapies reported that 6 weeks post-SCI is the minimum amount of time required for rodents to exhibit the molecular and histological characteristics of chronic SCI patients [37]. To optimize the rehabilitative strategy and maximize the therapeutic effect, the effects of regenerative rehabilitation on the chronically injured spinal cord and the underlying mechanisms must be delineated. It is even more important to clarify the effects of regenerative rehabilitation when performing fair and comparable preclinical studies because rehabilitation is sometimes so effective that it induces functional recovery of chronic SCI patients by itself [38]. Consequently, some clinical trials of stem cell treatment excluded patients who showed a possibility of recovery with rehabilitation [39] or did not receive any rehabilitative training [40]. A recent review reported that 18 out of 22 clinical studies of acute-to-subacute SCI and 17 out of 31 studies of chronic SCI published up to the middle of 2021 did not provide any details about rehabilitation protocols [41]. To develop regenerative rehabilitation and optimize rehabilitation, we should accurately elucidate the mechanisms of rehabilitation and assess whether recovery can be induced by combinatorial treatment with transplantation and rehabilitation, or by single treatments.

Very few researchers have reviewed regenerative rehabilitation targeting SCI, particularly in the chronic phase. Therefore, the present review aims to organize and summarize the mechanisms and effects of regenerative rehabilitation, including rehabilitative training and physical medicine modalities.

## 2. Regenerative Rehabilitation

In 2010, Ambrosio et al. first suggested the concept of regenerative rehabilitation as the optimized application of rehabilitation science to promote regenerative therapies [27]. In a broader sense, rehabilitation science and medicine cover treatments that incorporate mechanical stimuli, including rehabilitative training, tissue loading, stretching, joint mobilization, and traction; and physical stimuli, including electrical stimulation, magnetic stimulation, temperature gradients, and ultrasound stimulation [26]. However, it is not appropriate to classify electric acupuncture, which uses an electrical stimulus to enhance the effects of acupuncture, as rehabilitative because its principle is rooted in alternative medicine developed in China and surrounding countries. While regenerative rehabilitation initially focused on musculoskeletal functioning, the concept was subsequently changed to include neuronal recovery using stem cell therapies [25]. Rand and Ambrosio recently defined regenerative rehabilitation as “The application of rehabilitation protocols and principles together with regenerative medicine therapeutics toward the goal of optimizing functional recovery through tissue regeneration, remodeling, or repair.” Regenerative rehabilitation studies seek to investigate modifications of complex cell–cell and cell–matrix interactions induced by in vivo physical stimuli to achieve optimal functional outcomes secondary to regenerative treatments [26]. Tashiro et al. categorized regenerative rehabilitation for SCI as conditioning/reconditioning, functional training, and physical exercise, according to the molecular and behavioral mechanisms [41]. To our knowledge, no study has investigated the safety of regenerative rehabilitation, but no adverse effects have been observed to date.

The following rehabilitative training methods have been applied in combination with stem cell therapies to animal models of SCI regardless of chronicity: bipedal treadmill training with body-weight support [31,35], active quadrupedal training [29,30], passive quadrupedal treadmill training [42], inclined quadrupedal treadmill training [43], cycling exercise [32,33], passive rotation [44], swimming training [45] for thoracic cord injury models, and climbing training [46] and functional training with a forepaw-reaching task for cervical SCI models [47,48]. On the other hand, the following physical medicine modalities have been applied using apparatus resembling that in clinical use: intermittent repetitive transcranial magnetic stimulation (rTMS) [49], regular rTMS [50], epidural electrical stimulation (ESS) [46], and a diode continuous-wave laser [51].

It is noteworthy that the status of regenerative rehabilitation differs between clinical and preclinical studies in several respects. First, individuals with SCI routinely undergo rehabilitation in clinical settings and frequently continue rehabilitative training to preserve their joint range-of-motion, muscle strength, mobility, and activities of daily living. Second, rehabilitation is feasible in many clinical settings and is therefore used in combination with stem cell therapies in clinical trials. Third, it is relatively easy to propose appropriate training for patients according to their impairments because there is much knowledge of SCI rehabilitation, and rehabilitation therapists and doctors can receive immediate feedback from patients. By contrast, rehabilitation training is less feasible, very time-consuming, and expensive in preclinical studies [52], and laboratories investigating regenerative treatment often lack the capabilities to perform rehabilitative techniques. Moreover, the appropriate training and its load have not been structurally validated or standardized among research groups. Standardized protocols have only very recently been proposed for forelimb functional training and quadrupedal treadmill training in SCI model rodents [52,53]. Consequently, it is difficult to compare the results of preclinical studies incorporating regenerative rehabilitation.

## 3. Rehabilitative Treatments Targeting Chronic SCI

Various combinatorial strategies have been investigated to improve the effects of stem cell therapies on the refractory chronically injured spinal cord [9]. Although many clinical trials of stem cell therapy targeting chronic SCI have applied rehabilitation, only two studies from a single research group investigated the effects of combinatorial treatment with NS/PC transplantation and bipedal body-weight-supported treadmill training (BWSTT) in the field of basic research [31,44]. Tashiro et al. applied BWSTT for 1 week before and 8 weeks after transplantation at 49 days post-injury (DPI) in a mouse model with severe thoracic cord contusion. Combinatorial treadmill training restored GABAergic activity, synaptogenesis, and axonal regeneration, and reduced the quantity of pain-related calcitonin gene-related peptide-positive fibers in the lumbar enlargement. By contrast, NS/PC transplantation seemed to upregulate serotonergic activity, which may be related to increased central pattern generator (CPG) activity. Incorporation of rehabilitative training significantly increased neuronal differentiation without affecting cell survival. At the lesion epicenter, neither the cross-sectional area nor the fiber count passing through the lesion was restored secondary to any treatment, while transplantation slightly recovered both the myelinated area and latency of motor-evoked potentials. Although animals who received combinatorial treatment showed significant functional recovery with respect to motor and sensory function compared with control animals, there were no significant differences between the combinatorial treatment and single treatment groups. Thus, no additional significant functional recovery might be expected when stem cell therapy is delivered in combination with rehabilitation. Therefore, the authors concluded that a further additional treatment(s) needs to be applied in combination with stem cell therapies in patients with chronic SCI. While it is not uncommon to perform pretraining before the central intervention for habituation of experimental animals to the training apparatus in studies of subacute SCI [29,54], Tashiro et al. first incorporated pretraining to resolve disuse syndrome of animals with chronic SCI [14]. In summary, regenerative rehabilitation directly influences transplanted cells and promotes a plastic change in lumbar enlargement, but does not induce any remarkable histological change at the lesion epicenter of the chronically injured spinal cord [31,36].

On the other hand, the effects of rehabilitation on SCI according to its chronicity have not been determined due to a lack of preclinical studies [52,53]. While it is unrealistic for humans not to receive any rehabilitative treatment until the chronic phase of SCI, early initiation of training may prevent studies strictly of chronic SCI. Therefore, it remains controversial when regenerative rehabilitation should be started in SCI animals who receive stem cell treatment in the chronic phase. Two research groups partly proposed an answer by comparing different timings of combined rehabilitation initiation. Dugan et al. compared the effect of the same rehabilitation regimen initiated at 5 or 35 DPI in combination with late subacute GABAergic neural progenitor cell transplantation at 28 DPI. They observed suppression of inflammation and restoration of GABAergic activity in the whole spinal cord, leading to amelioration of heat hyperalgesia, cold allodynia, and tactile allodynia at both initiation timings [43]. While they did not perform stem cell therapy, Theisen et al. compared the effects of combined rehabilitation initiated at 5 or 35 DPI in a model that underwent peripheral nerve grafting at 42 DPI. There were no significant differences between the two initiation timings in terms of regenerating axons extending into peripheral nerve grafts [33]. These results indicate that the effects of rehabilitation are preserved regardless of when it is initiated.

## 4. Effects of Regenerative Rehabilitation

Distinct effects of regenerative rehabilitation on chronic SCI have been reported according to the lesion epicenter, transplanted cells, and lumbar enlargement. To delineate the specificity of regenerative rehabilitation in the chronic phase, we summarized the histological, biological, and physiological effects at various sites reported by studies of SCI in the acute-to-subacute and chronic phases. We conclude that most findings in every study of regenerative rehabilitation are independent, i.e., causal relationships are not clearly depicted because this research field is in its infancy. Therefore, we categorized the results according to the nature of the effects, including (1) the direct effect on transplanted stem cells, (2) the effect on the microstructure around the lesion including scar tissue, (3) the effect on the microstructure of spinal cord tissue distant from the lesion, and (4) the mechanisms underlying histological changes (Table 1, Table 2, Table 3 and Table 4). The second and third groups include histological features, represented by the types of neurons and neuronal fibers. By contrast, the fourth group is characterized by molecular changes, including amelioration of inflammation and upregulation of neurotrophic factors. Neurotrophic factors are frequently investigated in studies of regenerative rehabilitation targeting acute-to-subacute SCI, but have not been assessed in a chronic SCI model [29,43,49,50].

### 4.1. Direct Effect on Transplanted Stem Cells

It is frequently reported that combined rehabilitation can change the fate of cell grafts, particularly NS/PCs, which differentiate, proliferate, and migrate after grafting. Two research groups reported identical results showing that NS/PCs differentiated more into neurons and oligodendrocytes, together with a better cell survival rate, when treadmill training was performed in combination with stem cell therapy in rat models of subacute cervical and thoracic SCI [29,30]. Tashiro et al. subsequently reported an increase in neuronal differentiation without an effect on cell survival in a chronic contusive thoracic SCI mouse model [31]. On the other hand, Sun et al. found that combined rehabilitation did not promote astrocyte-like OEC activity in the subacute phase [35]. These results indicate that regenerative rehabilitation via rehabilitative training promotes differentiation into neurons and oligodendrocytes, not astrocytes, and that these effects decrease over time.

Similar results were reported in studies of physical medicine modalities both in vitro and in vivo. Guo et al. acutely transplanted human umbilical cord blood-derived MSCs (hUCB-MSCs) and applied intermittent rTMS composed of stimulation for 3 s and rest for 6 s at a frequency of 0.5 or 10 Hz. They reported increased proliferation of transplanted cells, consistent with facilitation of differentiation into neurons, oligodendrocytes, and astrocytes. The effect was greater in the high frequency (10 Hz) group [49]. Feng et al. performed bone marrow mesenchymal stem cell (BMSC) transplantation in combination with regular rTMS at 10 Hz, and found that rTMS promoted neuronal differentiation [50]. Although these studies do not report identical results, rTMS seems to increase neuronal differentiation. Due to the limited number of studies, it is difficult to discuss the different effects of cells derived from different sources.

### 4.2. Effect on the Microstructure around the Lesion

A massive microenvironmental change in the lesion epicenter restricts neuronal plasticity and the effects of stem cell therapies in various respects, and regenerative rehabilitation seems to attenuate these processes. Histological changes are observed around the lesion epicenter secondary to combined rehabilitation in individuals with acute-to-subacute SCI. Restoration of gross tissue volume, structure, and residual fibers is frequently reported when quadrupedal gait training is performed in combination with transplantation of NS/PCs [29,30] or bone marrow-derived cells (BMCs) [28]. Similarly, these changes were observed when rTMS was performed in combination with hUCB-MSC transplantation and when low-level laser treatment was performed in combination with human adipose tissue-derived stem cell (hASC) transplantation [49,51]. Myelination, formation of myelinated axons, and white matter sparing were reported when quadrupedal gait training was performed in combination with transplantation of NS/PCs [29,30] or BMCs [28]. Feng et al. reported that rTMS prevented apoptosis in damaged tissue when performed in combination with BMSC transplantation [50].

In addition to tissue-protective effects, spinal tract regeneration has also been reported. Guo et al. revealed that rTMS in combination with hUCB-MSC transplantation promoted regeneration of the corticospinal tract based on BDA tracing [49]. Younsi et al. showed that quadrupedal gait training in combination with NS/PC transplantation facilitated regeneration of reticulospinal and rubrospinal tracts based on quantification of fluorogold-positive axons in the red nucleus and reticular nucleus [30]. Beneficial effects in electrophysiological evaluation, such as restoration of the motor-evoked potential amplitude, have been reported along with tract regeneration, and such evaluation can also reflect reorganization of the motor system at the lower motoneuron level [30,49]. Sarveazad et al. reported that application of a diode continuous-wave laser in combination with hASC transplantation upregulated glutamic acid decarboxylase-65 and GABAB receptor 1 in the lesion epicenter [51]. These results suggest that regenerative rehabilitation modifies the microstructure of the lesion epicenter secondary to stem cell therapy. However, it is unknown if such effects are sustained until the chronic phase of SCI [31,36] or if regenerative rehabilitation can modify the features or process of fibrotic and glial scarring.

### 4.3. Effect on the Microstructure of Spinal Cord Tissue Distant from the Lesion

Plastic changes in microstructures distant from the lesion, represented by neural regeneration and neuronal circuit reorganization, are frequently reported as the main effects of regenerative rehabilitation. Rehabilitation enhances the activities of various neuronal subtypes. Serotonergic activity, assessed by serotonergic fiber innervation into the lumbar spinal cord, is increased secondary to quadrupedal treadmill training in combination with NS/PC transplantation in the acute/subacute phase [29]. Noradrenergic and dopaminergic neuronal plasticity, assessed by tyrosine hydroxylase-immunoreactivity at lumbar enlargement, is enhanced secondary to bipedal treadmill training in combination with OEC and Schwann cell transplantation in the subacute phase [35]. In addition, it is noteworthy that restoration of GABAergic activity was reported in both subacute and chronic models that underwent intensive quadrupedal treadmill or bipedal treadmill training in combination with NS/PC transplantation [31,36,43]. Synaptogenesis, axonal regeneration, and a decrease in pain-transmitting fibers were even reported in a chronic SCI model that underwent bipedal training and NS/PC transplantation [31,36]. Restoration of the motor-evoked potential amplitude in acute/subacute models following bipedal treadmill training and rTMS is indicative of reorganization of the lumbar spinal circuit and is also related to descending tract regeneration as previously described [34,49].

Sachdeva et al. [32] and Theisen et al. [33] revealed that cycling exercise in combination with peripheral nerve grafting outside the lesion enhanced regeneration of spinal axons into peripheral nerve grafts. A series of studies reported the same effect of regenerative rehabilitation upon early grafting with early rehabilitation, chronic grafting with early rehabilitation, and even chronic grafting with delayed rehabilitation [31,33]. Regenerative rehabilitation distant from the lesion may promote broad neural regeneration.

### 4.4. Mechanisms Underlying the Histological Changes

The molecular mechanisms that may induce the abovementioned histological changes can be grossly classified as trophic support and anti-inflammation. Various neurotrophic factors are upregulated secondary to rehabilitative training and physical medicine treatment. Brain-derived neurotrophic factor (BDNF), glial cell line-derived neurotrophic factor, insulin-like growth factor-1 (IGF-1), neurotrophin 3 (NT-3) [29], BDNF [43], and neurotrophin 4 [28] are reportedly upregulated via treadmill training. On the other hand, upregulation of BDNF and nerve growth factor by rTMS [50] and upregulation of basic fibroblast growth factor and epidermal growth factor (EGF) by intermittent rTMS [49] have also been reported.

A few studies revealed the involvement of other molecular mechanisms. Hwang et al. [29] revealed that suppression of reactive oxygen species/reactive nitrogen species by treadmill training attenuates cellular stresses induced downstream of IGF-1 signaling. This seems to be the only study that conclusively demonstrated the causal relationship of specific molecular events and their consequences upon regenerative rehabilitation. Feng et al. [50] suggested that suppression of neuronal apoptosis is related to downregulation of Raf/MEK/ERK signaling secondary to rTMS. Dugan et al. [39] showed that BDNF expression was reduced after SCI and restored secondary to quadrupedal treadmill training. BDNF contributes to upregulation of kalium-chloride cotransporter 2 in the dorsal horn, which moderates the hyperexcitable state of the local spinal network [55], leading to amelioration of allodynia and hyperalgesia.

Dugan et al. [43] reported the anti-inflammatory effects of intensive quadrupedal treadmill training. They demonstrated that expression of the anti-inflammatory marker IL4 was upregulated, and that of the proinflammatory cytokines tumor necrosis factor-alpha and interleukin 1β in cerebrospinal fluid was downregulated secondary to training.

**Figure 1:**
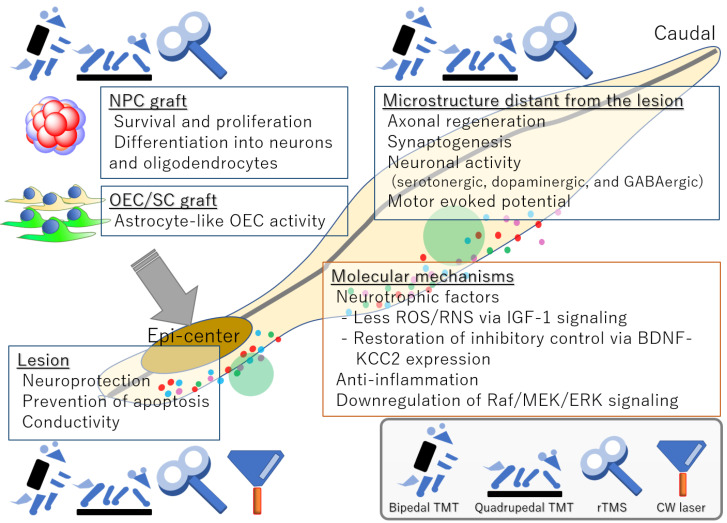
A schematic summary of the combinational effects of rehabilitation and regenerative treatment. BDNF: brain-derived neurotrophic factor; CW: continuous wave; IGF-1: insulin-like growth factor 1; KCC2: kalium-chloride cotransporter 2; OEC/SC: olfactory ensheathing cell/Schwann cell; ROS/RNS: reactive oxygen species/reactive nitrogen species; rTMS: repetitive transcranial magnetic stimulation; TMT: treadmill training.

Although there are only a few reports about the mechanisms underlying the changes induced by combinatorial use of treadmill training and stem cell therapy, such research will help to explore more effective approaches and prevent adverse effects [14]. Above mentioned mechanisms are summarized in Figure 1.

## 5. Further Combinatorial Therapies to Treat the Chronically Injured Spinal Cord

Regenerative rehabilitation elicits various beneficial effects on transplanted cell grafts and the host microstructure around the lesion and in distant areas. However, there is no evidence of its effects on fibrotic and glial scarring or around the lesion of the chronically injured spinal cord. This indicates that a further combinatorial treatment(s), such as medication, is needed to complement the effects of regenerative rehabilitation. It is important to consider if medication and training have overlapping effects because there is a concern that they do not elicit a synergistic effect [56]. Therefore, we will briefly overview combinatorial treatment with rehabilitation and medication. The combinatorial effects of the following agents with rehabilitative interventions on SCI have been investigated in preclinical studies: chondroitinase ABC (cABC) and keratanase II, which digest components of glial scars [57,58,59,60,61]; a semaphorin 3A inhibitor [60] and anti-Nogo-A antibody [57,61], which inhibit a neurite growth inhibitor expressed in the extracellular matrix during fibrotic scarring; granulocyte colony-stimulating factor, which suppresses inflammation at the lesion; quipazine and 8-OHDPAT [62,63,64,65], which are serotonin receptor agonists; and neurotrophic factors represented by BDNF, NT-3, hepatocyte growth factor, and a growth factor cocktail [58,66,67,68]. Cyproheptadine, a serotonin receptor antagonist, has also been applied to suppress spasticity [69].

Several studies reported significant combinatorial effects using cABC, which digests CSPG, in various experimental conditions, including intensive voluntary forepaw motor rehabilitation of rats with a dorsal corticospinal tract lesion [70], and quadrupedal gait training for rats with very severe SCI in the chronic phase [15]. Keratanase II, which digests keratan sulfate, is another candidate to treat glial scarring. Ishikawa et al. [59] demonstrated that cABC and keratanase II have equivalent effects on neurite growth and functional recovery. Zhang et al. [60] demonstrated a synergistic effect of a semaphorin 3A inhibitor and weight-supported bipedal treadmill training in spinalized rats in which a drug-containing silicon sheet similar to an artificial dura mater was implanted over the transected spinal lesion. On the other hand, Maier et al. [61] found that asynchronous combinatorial treatment with an anti-Nogo-A antibody and treadmill training did not elicit an additive effect, but increased erratic and dysfunctional stepping in rats with incomplete thoracic SCI. Zhao et al. [57] subsequently investigated a more practical treatment strategy in which treadmill training was used in combination with an anti-Nogo-A antibody and cABC. They applied the anti-Nogo-A antibody acutely and implemented rehabilitation after cessation of anti-Nogo-A antibody treatment according to a previous study by Maier et al. [61], which showed the discordant effect of an anti-Nogo-A antibody and treadmill training, and demonstrated that the combinatorial treatment regimen elicited an enhanced effect. While the effects on the chronically injured spinal cord have not been completely clarified, these agents, which modify the microenvironment of the lesion, may be promising candidates for combinatorial usage with regenerative rehabilitation and stem cell therapies.

Serotonin receptor agonists may enhance the neuromodulatory effect of EES. Quipazine, a non-selective 5-HT2A receptor agonist, facilitates motor function when used in combination with robotic locomotor training [62]. Courtine et al. reported a synergistic effect of EES at S1 plus L2 and the serotonergic agents quipazine and 8-OHDPAT, a 5-HT1A and 5-HT7 receptor agonist. They suggested that combinatorial use of quipazine and 8-OHDPAT elicits different effects on hindlimb functional recovery in rodents. Specifically, quipazine facilitates extension components, while 8-OHDPAT is more related to rhythmic movements [63]. This group further incorporated rehabilitation using a gravity-assisted bipedal gait robot and demonstrated improved locomotor recovery due to reinforcement of reticulospinal projections [64,65].

Neurotrophic factors, represented by NT-3 and BDNF, enhance the therapeutic effect of rehabilitation [67,71]. In addition, Alluin et al. [58] showed that the effect of combinatorial treatment with cABC and quadrupedal treadmill training could be strengthened using a growth factor cocktail comprising EGF, fibroblast growth factor, and platelet-derived growth factor-AA. However, it remains to be elucidated whether these pharmacological strategies targeting the CPG or training-induced effect itself can alter the influence of regenerative rehabilitation in combination with stem cell therapy on chronic SCI because glial and fibrotic scarring is a significant barrier that blocks the effect of stem cell therapy.

## 6. Conclusions

While studies have revealed the synergistic effects of regenerative rehabilitation with stem cell therapy on SCI, they seem insufficient to achieve significant functional recovery in the chronic phase. Preclinical studies have delineated the advantages and limitations of regenerative rehabilitation in inducing molecular and histological changes in the chronically injured spinal cord. Thus, it has been gradually revealed under which circumstances further combinatorial treatment with medication should be implemented. To establish a treatment regimen for robust functional recovery upon chronic SCI, further investigations are required of combinatorial treatment with stem cell therapy, regenerative rehabilitation, and medication.

## Figures and Tables

**Table 1:** Direct effects of transplanted cells.

Rehabilitative Training				
Study	Model	Grafting	Rehabilitation	Effect
Hwang et al. [29]	T9 moderate contusion rat	NPCs 7 DPI	Quadrupedal treadmill Initiated at 3 DPI	Transplanted cell survival Differentiation into neurons and oligodendrocytes Decrease in undifferentiated NPCs
Younsi et al. [30]	C6 moderate contusion rat	NPCs 10 DPI	Quadrupedal treadmill	Transplanted cell survival Differentiation into neurons and oligodendrocytes
Sun et al. [35]	T10 moderate contusion rat	OECs + SCs 14 DPI	Bipedal treadmill	Consistent astrocyte-like OEC activity
Tashiro et al. [31]	T9 severe contusion mouse	NS/PCs 49 DPI	Bipedal treadmill Initiated at 42 DPI	Unaffected transplanted cell survival Differentiation into neurons
**Physical Medicine Treatment**				
**Study**	**Model**	**Grafting**	**Rehabilitation**	**Effect**
Guo et al. [49]	T10 moderate contusion rat	hUCB-MSCs 2 DPI	Intermittent 0.5 or 10 Hz rTMS	While both conditions are effective, greater at 10 Hz Transplanted cell proliferation Differentiation into neurons, oligodendrocytes, and astrocyte (non-significant)
Feng et al. [50]	T10 moderate contusion rat	BMSCs 7 DPI	10 Hz rTMS Initiated at 1 DPI	Differentiation into neurons

BMSCs: bone marrow mesenchymal stem cells; DPI: days post-injury; hUCB: human umbilical cord blood; MSCs: mesenchymal stem cells; NPCs: neural precursor cells; NS/PCs: neural stem/precursor cells; OECs: olfactory ensheathing cells; rTMS: repetitive transcranial magnetic stimulation; SCs: Schwann cells.

**Table 2:** Effects on the microstructure around the lesion.

Rehabilitative Training				
Study	Model	Grafting	Rehabilitation	Effect
Takeoka et al. [34]	T9 transection rat	OEG 0 DPI	Manual stepping and bipedal treadmill	MEP amplitude restoration
Hwang et al. [29]	T9 moderate contusion rat	NPCs 7 DPI	Quadrupedal treadmill Initiated at 3 DPI	Tissue sparing Myelination
Massoto et al. [28]	T9 clip contusion mouse	BMCs 7 DPI	Quadrupedal treadmill Initiated at 14 DPI	White matter and myelinated fiber sparing Less micro-cavitation Fewer degenerating fibers
Younsi et al. [30]	C6 moderate contusion rat	NPCs 10 DPI	Quadrupedal treadmill	Tissue and myelination area sparing Regeneration of descending tracts
**Physical Medicine Treatment**				
**Study**	**Model**	**Grafting**	**Rehabilitation**	**Effect**
Guo et al. [49]	T10 moderate contusion rat	hUCB-MSCs 2 DPI	Intermittent 0.5 or 10 Hz rTMS	While both conditions are effective, greater at 10 Hz Tissue sparing CST regeneration assessed by BDA tracing MEP amplitude restoration
Feng et al. [50]	T10 moderate contusion rat	BMSCs 7 DPI	10 Hz rTMS Initiated at 1 DPI	Fewer apoptotic cells
Sarveazad et al. [51]	T13-L1 clip compression rat	hASCs 7 DPI	Diode CW laser (660 nm wavelength at 100 mW) Initiated at 0 DPI and continued for 2 weeks	Upregulation of GAD65 and GABA_B_ receptor 1 expression More axons around the cavity

BMCs: bone marrow cells; BMSCs: bone marrow mesenchymal stem cells; CST: corticospinal tract; CW: continuous wave; DPI: days post-injury; GAD: glutamic acid decarboxylase; hASCs: human adipose-derived stem cells; hUCB: human umbilical cord blood; MEP: motor-evoked potential; MSCs: mesenchymal stem cells; NPCs: neural precursor cells; OEG: olfactory ensheathing glia; rTMS: repetitive transcranial magnetic stimulation.

**Table 3:** Effects on the microstructure of spinal cord tissue distant from the lesion.

Rehabilitative Training				
Study	Model	Grafting	Rehabilitation	Effect
Takeoka et al. [34]	T9 transection rat	OEG 0 DPI	Manual stepping and bipedal treadmill	MEP amplitude restoration
Hwang et al. [29]	T9 moderate contusion rat	NPCs 7 DPI	Quadrupedal treadmill Initiated at 3 DPI	Innervation of serotonergic fibers into the lumbar spinal cord
Sun et al. [35]	T10 moderate contusion rat	OECs + SCs 14 DPI	Bipedal treadmill	Dopaminergic tyrosine hydroxylase-positive neurons
Dugan et al. [43]	T6/7 clip contusion rat	GABAergic NPCs 28 DPI	Quadrupedal inclined treadmill Early (5 DPI) or delayed (35 DPI) initiation	Restoration of GABAergic activity, especially in the dorsal horn
Tashiro et al. [31]	T9 severe contusion mouse	NS/PCs 49 DPI	Bipedal treadmill Initiated at 42 DPI	Synaptogenesis Axonal regeneration GABAergic activity at lamina V–VII
Tashiro et al. [36]	T9 severe contusion mouse	NS/PCs 49 DPI	Bipedal treadmill Initiated at 42 DPI	Reduction of pain transmission fibers GABAergic activity at the dorsal horn
**Physical Medicine Treatment**				
**Study**	**Model**	**Grafting**	**Rehabilitation**	**Effect**
Guo et al. [49]	T10 moderate contusion rat	hUCB-MSCs 2 DPI	Intermittent 0.5 or 10 Hz rTMS	While both conditions are effective, greater at 10 Hz CST regeneration assessed by BDA tracing MEP amplitude restoration

CST: corticospinal tract; DPI: days post-injury; hUCB: human umbilical cord blood; MEP: motor-evoked potential; MSCs: mesenchymal stem cells; NPCs: neural precursor cells; NS/PCs: neural stem/precursor cells; OECs/OEG: olfactory ensheathing cells/glia; rTMS: repetitive transcranial magnetic stimulation; SCs: Schwann cells.

**Table 4:** Further mechanisms underlying the effects of regenerative rehabilitation.

Rehabilitative Training				
Study	Model	Grafting	Rehabilitation	Effect
Hwang et al. [29]	T9 moderate contusion rat	NPCs 7 DPI	Quadrupedal treadmillInitiated at 3 DPI	BDNF, GDNF, IGF-1, and NT-3 detected by ELISAs and immunohistochemistry at the lesion Suppression of ROS/RNS, leading to attenuation of cellular stresses, induced by IGF-1 signaling
Massoto et al. [28]	T9 clip contusion mouse	BMCs 7 DPI	Quadrupedal treadmill Initiated at 14 DPI	NT-4 immunoreactivity at the lesion
Dugan et al. [43]	T6/7 clip contusion rat	GABAergic NPCs 28 DPI	Quadrupedal inclined treadmill Early (5 DPI) or delayed (35 DPI) initiation	Anti-inflammatory marker (IL4) expression by delayed training Reduced levels of proinflammatory markers (TNFα and IL1β) in CSF BDNF expression in the lumbar spinal cord Restoration of KCC2 expression
**Physical Medicine Treatment**				
**Study**	**Model**	**Grafting**	**Rehabilitation**	**Effect**
Guo et al. [49]	T10 moderate contusion rat	hUCB-MSCs 2 DPI	Intermittent 0.5 or 10 Hz rTMS	While both conditions are effective, greater at 10 Hz bFGF and EGF expression at the lesion
Feng et al. [50]	T10 moderate contusion rat	BMSCs 7 DPI	10 Hz rTMS Initiated at 1 DPI	BDNF and NGF expression Downregulation of Raf/MEK/ERK signaling related to neuronal apoptosis

BDNF: brain-derived neurotrophic factor; bFGF: basic fibroblast growth factor; BMCs: bone marrow cells; BMSCs: bone marrow mesenchymal stem cells; CSF: cerebrospinal fluid; DPI: days post-injury; EGF: epidermal growth factor; GDNF: glial cell line-derived neurotrophic factor; hUCB: human umbilical cord blood; IGF-1: insulin-like growth factor 1; IL1β: interleukin 1β; IL4: interleukin 4; KCC2: kalium (potassium) chloride cotransporter 2; MSCs: mesenchymal stem cells; NGF: nerve growth factor; NPCs: neural precursor cells; NT-3: neurotrophin 3; NT-4: neurotrophin 4; ROS/RNS: reactive oxygen species/reactive nitrogen species; rTMS: repetitive transcranial magnetic stimulation; TNFα: tumor necrosis factor α.

## Data Availability

Not applicable.
